# Thyroid Function in Obese Children with Non-Alcoholic Fatty Liver Disease

**DOI:** 10.4274/jcrpe.1488

**Published:** 2014-09-05

**Authors:** Hüseyin Bilgin, Özgür Pirgon

**Affiliations:** 1 Turgut Özal University, Department of Pediatrics, Division of Neonatology, Ankara, Turkey; 2 Süleyman Demirel University, Departments of Pediatrics, Division of Pediatric Endocrinology and Diabetes, Isparta, Turkey

**Keywords:** thyroid function, obesity, non-alcoholic fatty liver, insulin resistance

## Abstract

**Ob­jec­ti­ve:** To investigate the relationships between thyroid function and metabolic risk factors in obese adolescents with non-alcoholic fatty liver disease (NAFLD).

**Methods:** One hundred sixty obese adolescents and 40 control subjects were enrolled in the study. The obese subjects were divided into two groups based on presence or absence of liver steatosis (NAFLD group and non-NAFLD group). Serum samples were assayed for glucose, insulin, cholesterol, alanine aminotransferase, aspartate aminotransferase, free thyroxine (fT4), free triiodothyronine (fT3) and thyroid-stimulating hormone (TSH). The ratio of fT3 to fT4 was evaluated as an indirect index of deiodinase activity. Insulin resistance was evaluated by homeostasis model assessment (HOMA-IR) from fasting samples.

**Results:** NAFLD and non-NAFLD groups had slightly higher fasting blood glucose values than the control group. Fasting insulin levels in the NAFLD group were significantly higher than those in the non-NAFLD and control groups. The NAFLD group had significantly greater HOMA-IR values compared with the non-NAFLD group and also with the control group. The NAFLD group had significantly higher fT3/fT4 ratio values compared to both non-NAFLD and control groups. fT3/fT4 was positively correlated with serum insulin levels in the NAFLD group.

**Conclusion:** This study showed that obese adolescents with hepatosteatosis had elevated values for fT3/fT4 ratio. This finding suggested a high conversion of T4 to T3 due to increased deiodinase activity as a compensatory mechanism for fat accumulation.

## INTRODUCTION

Obesity is considered a worldwide health problem and its prevalence is known to increase steadily and dramatically all over the world (1). In parallel with epidemic obesity, non-alcoholic fatty liver disease (NAFLD) has also been increasingly recognized worldwide in the last decade. NAFLD encompasses a histological spectrum from isolated hepatic steatosis to steatosis with inflammation, cell injury and possible fibrosis. Some cases may progress to cirrhosis, portal hypertension and consequently, to liver-related death in early adulthood (2,3). Obesity affects hypothalamic-pituitary-thyroid axis directly or indirectly leading to alterations in thyroid function tests.

Subclinical hypothyroidism (SH) is a preliminary stage of overt hypothyroidism, defined as a mild elevation of thyroid-stimulating hormone (TSH) level in a patient with normal serum thyroxine (T4) level. SH has been found to be associated with hyperlipidemia, insulin resistance and obesity in adults. However, to our knowledge, there are no reported studies on the interaction of SH and free triiodothyronine (fT3) to free T4 (fT4) ratio in obese adolescents with NAFLD in comparison with age-matched healthy controls. This present study was undertaken to investigate, also considering the effect of metabolic risk factors, the relationships between SH and fT3/fT4 ratio in obese adolescents with NAFLD.

## METHODS

One hundred sixty consecutive obese adolescents (81 girls and 79 boys, mean age: 12.5±2.1 years, age range 10-15) recruited from pediatric patients admitted to our department of pediatric endocrinology between April 2010 and June 2011 were included in the study. The obese subjects were divided into two groups based on presence or absence of liver steatosis with high transaminases. The non-NAFLD group included 80 obese patients without liver steatosis (40 girls and 40 boys, mean age: 12.4±2.1 years, age range 10-15). The NAFLD group included 80 obese patients with liver steatosis (41 girls and 39 boys, mean age: 12.6±2.1 years, age range 10-15). Lean adolescents (21 girls and 19 boys, mean 1.9±2.5 years, age range 9-15) were enrolled in the study by selection from a group of non-obese healthy adolescents who attended the hospital for reasons other than infection, malignancy and without a history of thyroid hormone therapy or use of drugs causing insulin resistance. Exclusion criteria were hepatic virus infections (hepatitis A, B, C, D, E and G, cytomegalovirus and Epstein-Barr virus), alcohol consumption, history of parenteral nutrition and use of drugs known to induce steatosis or affect thyroid functions (e.g. valproate, amiodarone, thyroid hormones, propylthiouracil or prednisone). Autoimmune and metabolic liver disease, Wilson’s disease and α-1-antitrypsin-associated liver disease were ruled out using standard clinical, laboratory and histological criteria. Family history for obesity and diabetes were obtained by questionnaires. None of the patients had a family history of diabetes. The study protocols were approved by the institutional review board of Konya Training and Research Hospital. Signed informed consent forms were obtained from the parents of the children. Height and weight were measured with an empty bladder in postabsorptive conditions. Height was measured to the nearest 0.5 cm, without shoes, back against the wall tape, eyes looking straight ahead, with a right-angled triangle resting on the scalp and against the wall. Weight was measured with a lever balance, to the nearest 100 g, without shoes, in light undergarments. Body mass index (BMI) was calculated as weight (in kilograms) divided by height (in meters squared). Patients with a BMI of ≥95th percentile according to reference curves for Turkish children and adolescents were accepted as obese (4). The pubertal development stage of the patients was assessed by a single pediatric endocrinologist using the criteria of Tanner stages. Sexual maturation stage was 2 or greater in all patients (Tanner stages 2-4). After the child had rested for at least 5 minutes and was in a sitting position, diastolic and systolic pressure (mmHg) measurements were taken using a mercury-gravity manometer and a cuff appropriate for body size. Blood samples for estimation of serum glucose, insulin levels and other parameters were collected between 08:00 and 10:00 a.m., following a 12-h overnight fasting period. Serum concentrations of total cholesterol, high-density lipoprotein cholesterol (HDL-cholesterol) and triglycerides were measured using routine enzymatic methods with Abbott Aeroset (USA) Analyzer. Value of low-density lipoprotein cholesterol (LDL-cholesterol) was calculated using Friedewald equation. In addition to standard liver function tests [alanine aminotransferase (ALT), aspartate aminotransferase (AST)], serum levels of calcium, phosphate, magnesium, alkaline phosphatase and creatinine were measured on the same day with an auto analyzer. Glucose was determined by the hexokinase method. Serum insulin levels were measured by an Immulite immunoassay (Siemens Diagnostics, USA). Insulin sensitivity index was derived from fasting blood samples. The homeostasis model assessment of insulin resistance (HOMA-IR) was calculated as fasting insulin concentration (µU/mL) × fasting glucose concentration (mmol/L)/22.5. A HOMA-IR value greater than 3.16 was considered indicative of insulin resistance (5). Metabolic syndrome was defined according to the modified World Health Organization (WHO) criteria adapted for children. Subjects were diagnosed as having metabolic syndrome if they met 3 of WHO criteria (6). Serum samples were assayed for fT4, fT3 and TSH levels using an automated chemiluminescence assay system (Advia Centaur XP). Normal values were as follows: fT3 3.5-6.4 pmol/L; fT4 9.54-23.09 pmol/L; TSH 0.35-4 µIU/L. SH was defined as fT4 at the normal range and increased TSH levels. The ratio fT3 to fT4 (fT3/fT4) was calculated as an indirect index of deiodinase activity (7). Autoantibodies against thyroid peroxidase (TPO-Ab) and against thyroglobulin (TG-Ab) were detected by radioimmunoassay. Patients with TPO-Ab and TG-Ab levels higher than 60 IU/mL, suggesting autoimmune thyroiditis, were excluded. Liver ultrasonography for assessment of liver steatosis was performed in all subjects by a trained operator who was blinded to all clinical and laboratory characteristics of the participants, using a General Electric Logic 9 (MI, USA) machine, equipped with 7.5 MHz probes in younger children and 5 MHz in larger or markedly obese children and adolescents. The diagnosis of NAFLD is usually based on mild elevations in liver tests noted during a blood test and liver ultrasonography results in an overweight or obese child. In the present study, all obese patients with NAFLD had high ALT levels defined as >30 U/L and the diagnosis of NAFLD was based on elevated ALT levels (>30 U/L) (8) confirmed with abnormal liver ultrasonography results as defined by Tominaga et al (9). Although liver ultrasonography can estimate neither fibrosis nor inflammation, it has a sensitivity of 89% and a specificity of 93% for detecting histological steatosis (10). All patients with abnormal high transaminases and abnormal liver ultrasonography results were screened for other liver conditions (hepatitis B surface antigen, hepatitis C antibody, prothrombin time, serum iron level, total iron binding capacity, ferritin and antinuclear antibodies) which were all negative.

**Statistical Analysis**

Mean and standard deviations (SD) were used as descriptive statistics. Differences in the means of variables were tested using both parametric and nonparametric tests depending on the distribution of the variables. The correlations among numerical data were analyzed by the Pearson’s correlation coefficient (r). To analyze the associations between categorical variables, Spearman’s rank correlation coefficient was used. Comparison between groups was performed using ANOVA (post hoc: Bonferroni). Regression analysis was performed by multivariate stepwise regression. A probability value of <0.05 was considered significant. The SPSS version 14 (SPSS, Chicago, IL, USA) was used for statistical analyses. Statistical Analysis Mean and standard deviations (SD) were used as descriptive statistics. Differences in the means of variables were tested using both parametric and nonparametric tests depending on the distribution of the variables. The correlations among numerical data were analyzed by the Pearson’s correlation coefficient (r). To analyze the associations between categorical variables, Spearman’s rank correlation coefficient was used. Comparison between groups was performed using ANOVA (post hoc: Bonferroni). Regression analysis was performed by multivariate stepwise regression. A probability value of <0.05 was considered significant. The SPSS version 14 (SPSS, Chicago, IL, USA) was used for statistical analyses. 

## RESULTS

Table 1 shows the demographic, anthropometric, physical and biochemical findings in the lean group and in the obese groups with and without NAFLD. Age, height and gender ratios were similar in all three groups.

BMI-SDS was significantly higher in the NAFLD and non-NAFLD groups compared to the control group (p: 0.001, p: 0.001, respectively). However, there was no significant difference between non-NAFLD and NAFLD groups for BMI-SDS (p: 0.12). While the obese groups showed no significant differences in terms of diastolic blood pressure (p: 0.05), both obese groups had significantly higher systolic blood pressure than the control group (p: 0.01, p: 0.01, respectively).

HDL cholesterol levels were higher in the non-NAFLD group than in the NAFLD group (p: 0.02). However, the control group had significantly higher HDL cholesterol levels compared to both NAFLD and non-NAFLD groups (p: 0.001, p: 0.001, respectively). No significant differences were found between NAFLD and non-NAFLD groups for other lipid levels. There were no significant differences in terms of triglycerides, total cholesterol and LDL levels between the non-NAFLD and lean groups. AST and ALT levels were higher in the non-NAFLD group than in the control group (p: 0.02, p: 0.03, respectively). However, the NAFLD group had significantly higher AST and ALT levels compared to both non-NAFLD and control groups.

NAFLD and non-NAFLD groups, as compared with the control group, were found to have slightly higher fasting blood glucose levels, but the differences were not statistically significant (p>0.05). The non-NAFLD group had significantly higher fasting insulin levels than the lean group (p: 0.001). However, the NAFLD group had significantly elevated fasting insulin levels compared to both control and non-NAFLD groups (p: 0.001, p: 0.01, respectively). The NAFLD group had significantly greater HOMA-IR values compared to the non-NAFLD group (p: 0.01) and to the control group (p: 0.001).

**Figure 1:**
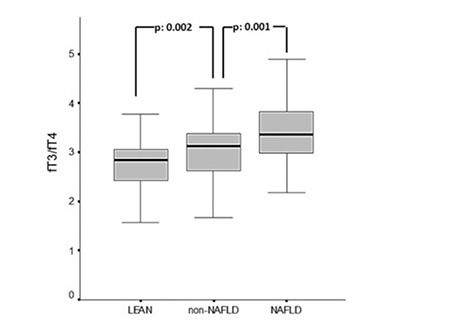
Comparison of fT3/fT4 ratio values among the NAFLD, non-NAFLD and lean groups. Mean values were 2.73±0.46, 3.08±0.61 and 3.48±0.75, respectivelyfT3: free triiodothyronine, fT4: free thyroxine, NAFLD: non-alcoholoc fatty liver disease

The control group had significantly greater fT4 values than the non-NAFLD group and also the NAFLD group, but fT4 levels were within the normal range in all groups. The NAFLD group had significantly higher levels of fT3 than the non-NAFLD and control groups (p: 0.001, p: 0.001, respectively). There was no statistically significant difference between the groups for TSH levels, but nineteen patients in the NAFLD group had elevated TSH levels. fT3/fT4 ratio was higher in the non-NAFLD group than in the control group (p: 0.002). However, the NAFLD group had significantly higher fT3/fT4 ratio compared to both non-NAFLD and control groups (p: 0.001, p: 0.001, respectively) (Figure 1).

Thyroid ultrasonography findings were reported as normal in all subjects.

To establish the prevalence of metabolic syndrome in the NAFLD and non-NAFLD groups, the obese groups were further classified into those with and without metabolic syndrome. The proportion of patients who had metabolic syndrome was 65.0% (52/80) in the NAFLD obese group, while this proportion was 22.5% (18/80) in the non-NAFLD obese group.

In the NAFLD group, fT3/fT4 was positively correlated with insulin levels (r: 0.54, p: 0.04) and negatively correlated with diastolic blood pressure values (r: -0.23, p: 0.03) (Table 2). fT3/fT4 ratio was positively correlated with fasting blood glucose (r: 0.64, p: 0.003), insulin (r: 0.52, p: 0.02) and HOMA-IR (r: 0.58, p: 0.008) in the NAFLD group with elevated TSH levels (TSH >4) (Table 3).

## DISCUSSION

To the best of our knowledge, our study is the first to evaluate the associations between fT3/fT4 ratio and NAFLD, which also includes a comparison with lean healthy controls. The findings of this study demonstrated that fT3/fT4 ratio was higher in obese adolescents with NAFLD compared to both non-NAFLD obese and lean control groups, suggesting a higher conversion from fT4 to fT3 due to increased deiodinase activity as a compensatory mechanism for fat accumulation to improve energy expenditure. Furthermore, we found positive associations among fT3/fT4 ratio, insulin and HOMA-IR. We emphasized that presence of NAFLD in an obese adolescent has a harmful effect on thyroid function and is also correlated with enhanced insulin resistance.

Little is known about the significance of thyroid function abnormalities in obesity. However, since these abnormalities often normalize with weight loss, they seem to be a reversible consequence of the weight status. In some studies, a correlation between TSH and/or thyroid hormones and BMI has been reported in adult obese subjects (11), but this relationship has not been shown in other studies (12). Stichel et al (13) reported an increased frequency of elevated TSH concentrations (7.5% above the upper normal limit of 4 U/L) in 290 obese children, with T3 concentrations significantly higher than in the control group (but within the normal range) and with normal T4. Reinehr and Andler (14) reported moderately increased peripheral thyroid hormones (T3, T4) and TSH levels in obese children. According to these authors, T3, T4 and TSH correlated with the degree of overweight but not with leptin or lipid levels. They also found that a reduction in overweight caused a significant decrease in T3, T4 and leptin serum concentrations, with no significant changes in TSH. In a more recent study, they reported that TSH and fT3, but not fT4, were significantly increased in obese children and that both were correlated with BMI (15).

Hepatic injury develops as hepatocytes and their anti-oxidant defenses are overwhelmed by oxidative stress as appears to be the case in NAFLD. Oxidative stress increases hepatic steatosis via lipid peroxidation increase and released cytokines lead to more mitochondrial damage (16,17). Mitochondrial injury can lead to mutation and loss of mitochondrial DNA (18). Since ATP is critical for maintaining cellular integrity, its depletion may predispose to hepatocellular injury (19). Mitochondrial ATP is a source of cyclic AMP, which is a second messenger for lots of hormones including TSH and TRH. With the depletion of ATP, the production of the second messenger c-AMP by adenylate cyclase decreases. Liver cells are one of the main targets of thyroid hormones and with depletion of cyclic AMP, the action of these hormones in hepatocytes would be affected. The clinical result is hepatocyte unresponsiveness to thyroid hormones and an increase in TSH level to overcome this decrease.

Progressive fat accumulation was associated with a parallel increase in TSH and fT3 levels irrespective of insulin sensitivity and metabolic parameters and a positive association has been reported between the fT3 to fT4 ratio and both waist circumference and BMI in obese women aged 18-68 years (7). This finding suggests a high conversion of T4 to T3 in patients with central fat obesity due to increased deiodinase activity as a compensatory mechanism for fat accumulation to improve energy expenditure (7). The study by Targher et al (20) involving a large cohort of unselected adult outpatients was the first to report a strong association between thyroid function tests and serum liver enzyme activity concentrations. In particular, Targher et al (20) found a significant positive relationship between serum TSH, ALT and GGT activities throughout the normal and high TSH ranges and a similar inverse relationship between fT4 and serum liver enzyme activity concentrations. Pacifico et al (21) reported that serum TSH concentrations were significantly higher in obese children with fatty liver as compared to children with no fatty liver. On the other hand, children with hepatic steatosis did not differ significantly from those with no hepatic steatosis with respect to fT3 and fT4. Hizli et al (22) found that SH frequency in obese children with fatty liver is not higher than that in patients without fatty liver.

In conclusion, the results of this study show that fT3/fT4 ratio values are elevated in obese adolescents with hepatosteatosis, suggesting a high conversion of T4 to T3 possibly due to increased deiodinase activity as a compensatory mechanism for fat accumulation. Our findings also point to a relationship between fT3/fT4 ratio and the severity of insulin resistance. Detection of a high fT3/fT4 ratio in an obese adolescent should alert the clinician to a possibility of NAFLD and insulin resistance.

**Ethical Conduct of Research**

The authors state that they have obtained appropriate institutional review board approval and have followed the principles outlined in the Declaration of Helsinki for all human or animal experimental investigations. Informed consent has been obtained from the parents of the participants.

## Figures and Tables

**Table 1:**
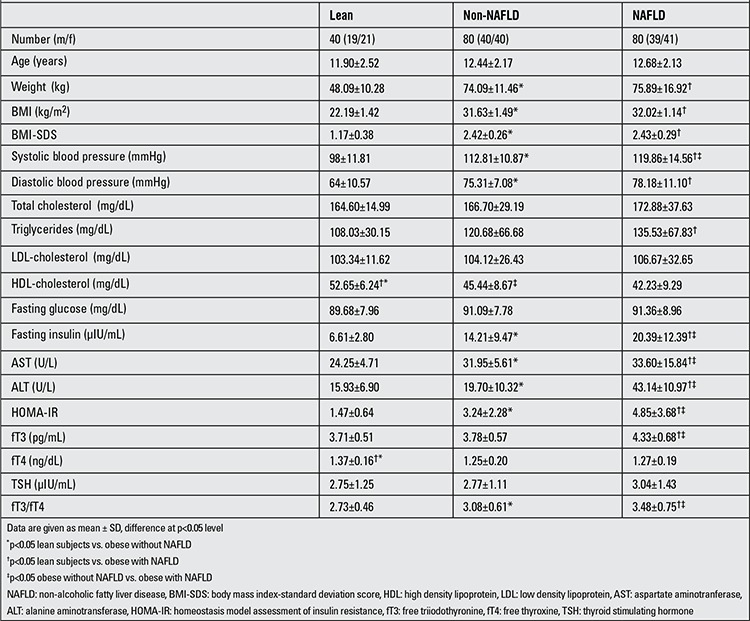
Demographic, anthropometric and other characteristics of lean and obese children with and without NAFLD

**Table 2:**
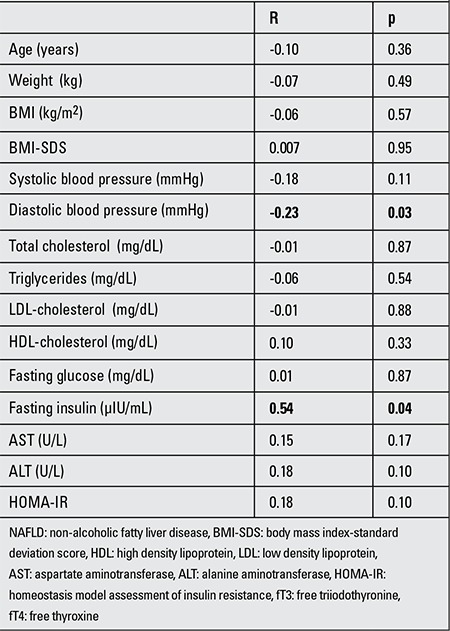
Pearson’s correlations of fT3/fT4 ratio with metabolic parameters and insulin sensitivity measurement in obese children with NAFLD

**Table 3:**
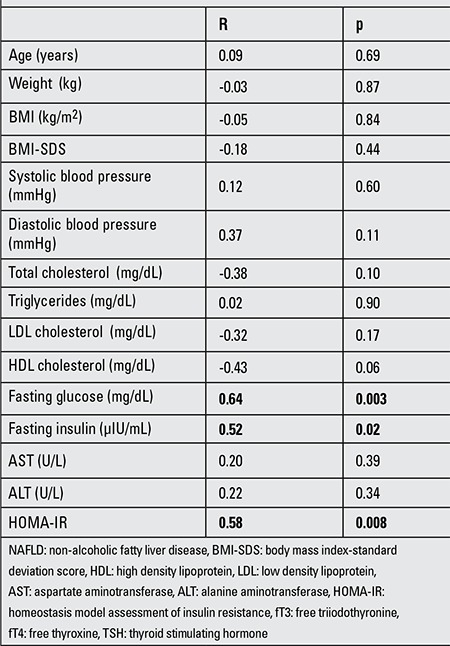
Pearson’s correlations of fT3/fT4 ratio with metabolic parameters and insulin sensitivity measurement in the NAFLD group with elevated TSH levels
